# The Interplay between Participatory Health Research and Implementation Research: Canadian Research Funding Perspectives

**DOI:** 10.1155/2018/1519402

**Published:** 2018-05-14

**Authors:** Erica Di Ruggiero, Nancy Edwards

**Affiliations:** ^1^Dalla Lana School of Public Health, University of Toronto, Health Sciences Building, 155 College Street, Suite 408, Toronto, ON, Canada M5T 3M7; ^2^School of Nursing, Faculty of Health Sciences, University of Ottawa, 1 Stewart Street, Room 205, Ottawa, ON, Canada K1N 6N5

## Abstract

**Objectives:**

The objective of this paper is to investigate what participatory health research (PHR) can offer implementation research (IR) and vice versa and discuss what health research funders can do to foster the intersection of both fields.

**Methods:**

We contrast points of divergence and convergence between IR and PHR. We reflect on whether community engagement is necessary and on the unintended consequences of participation in IR. We describe how a research funder can incentivize PHR in IR.

**Results:**

Participation is encouraged in IR but the nuances of who is involved merit greater attention in IR. PHR and IR differ in emphasis placed on the scale-up of the intervention. However, they share a common interest in generating real-world contextually relevant evidence.

**Conclusions:**

We need to assess whether and how funding practices influence researchers in how they undertake PHR. Researchers need to better account for participatory approaches to ensure that any potentially harmful consequences are described (and better understood so they can be mitigated in the future) and elucidate the ways in which these processes do or do not enable implementation and scale-up of interventions in IR.

## 1. Introduction

Increasingly, researchers and research funders around the globe are turning their attention to funding and conducting implementation research (or science). This growing trend is in response to a need for real-world and contextually sensitive evidence to respond to and solve implementation problems facing policy-makers, practitioners, communities, and other social actors working in sectors such as health [1, 2]. Lack of community readiness or engagement and a poor understanding of the sociopolitical and cultural contexts in which interventions are implemented are among the reasons cited for this limited progress in using evidence in policy and practice settings [3, 4].

“Implementation science examines what works, for whom, under what contextual circumstances, and whether interventions are scalable in equitable ways. The intervention includes the “what”—[for instance, a policy or community program]—as well as the “how”—the implementation activities that are required to achieve full (equitable) coverage of …the intervention” [2]. Like with any emerging field, a plurality of research methods and theories are expected to contribute to the further conceptualization, design, and conduct of implementation research studies. In recent decades, interest in participatory health research (PHR) has been steadily rising, in part due to increased funding incentives for academics to cocreate knowledge and align research with the real-world needs and interests of different actors with the promise of greater applicability and impact of the research for communities, policy-makers, and practitioners [5–7].

In light of such trends, research funders are responding. The Canadian Institutes of Health Research (CIHR), Canada's premier health research funding agency, supports research that is either investigator-led or priority-driven across the four health research pillars/themes (biomedical, clinical, and health services and social, cultural, environmental, and population health) [8]. While PHR is generally applicable to three of the four pillars of health research at CIHR, this article focuses on research funding initiatives addressing two of the four (health services and social, cultural, environmental, and population health). CIHR has, over the last five years, stepped up its contributions to implementation research (IR) through its investments in global health research (e.g., maternal and child health, chronic diseases) [9], Pathways to Health Equity for Aboriginal Peoples [10], HIV/AIDs [11], and various health systems and patient-oriented research initiatives to name but a few [12]. CIHR has also declared its commitment to citizen engagement in health research, which it defines as the meaningful involvement of individual citizens in activities such as research priority-setting, planning, governance, and peer review to enhance the relevance and application of research into practice and policy [13].

## 2. Methods

In this commentary-style article, we pose the following central questions: what does participatory health research (PHR) have to offer implementation research (IR) and what does IR have to offer PHR? We start by looking at how PHR and IR diverge and how they intersect or converge with respect to participatory approaches. Then, we briefly consider the following questions in relation to implementation research: Who are the “participants” in IR studies and is community engagement always necessary in IR? What are the possible unintended consequences of participatory processes in the context of IR studies? Using illustrative examples from recently funded research initiatives, we also discuss what roles health research funders can play in fostering the intersection of participatory health research and implementation research and consider how this would advance research in both fields.

## 3. Results

### 3.1. What Does Participatory Health Research (PHR) Have to Offer Implementation Research (IR), and What Does IR Have to Offer PHR? How Do They Intersect and Diverge from Each Other?

While it is well beyond the scope of this paper to review all possible theoretical and methodological approaches that underpin PHR (refer to other papers in this supplement), suffice it to say that PHR approaches hold some common core principles. These include meaningful community engagement and cocreation of knowledge that accounts for different ways of knowing, commitment to social change extending beyond the generation of knowledge, grounding in participants' lived experiences in the local context, and reflexivity [14, 15]. PHR has been defined as a “transformative research paradigm that bridges the gap between science and practice through community engagement and social action to increase health equity” [16, p.40]. It is also a field of research that goes by different names (community-based research, action research, community-based participatory research, participatory methods, etc.), reflecting different country and ideological and disciplinary traditions [17].

### 3.2. Points of Divergence between PHR and IR

Implementation researchers are encouraged to be more explicit about the “nature” of the intervention (which should also include who defines what “it” is), what underlying sociostructural determinants are at play, and how these need to be considered in planning for both horizontal and vertical scale-up of the intervention [2]. The starting point in IR is usually innovations or interventions with some level of demonstrated effectiveness. While participation is certainly encouraged in IR, we argue that the nuances of participatory engagement processes and related measures have not received sufficient attention in IR [18]. This matters because participant characteristics and their structural influences can affect implementation effectiveness [19]. The focus is generally on the participation of those responsible for delivering or implementing the innovation or intervention. This tendency reflects some of the roots of implementation research (science), which stem from quality assurance initiatives and their related focus on those formally responsible for health services delivery [1, 2]. The quality assurance approach involves the plan-do-study-act cycle but PHR and IR diverge with respect to who is primarily involved in this cycle of reflexivity [20].

A second point of divergence concerns scale-up of the intervention. While PHR is mainly focused on the codesign of research questions, participation in implementation, and analysis processes, implementation research (or science) is usually concerned with approaches that will enhance scale-up of effective interventions. A key difference between PHR and IR is therefore the extent of emphasis on scale-up, stemming in part from the “who” is participating in IR (i.e., usually actors delivering the intervention). There are in fact many examples of PHR where the potential scalability of the innovation or intervention and the scalable unit are simply not considered throughout the research process [21].

### 3.3. Points of Intersection between PHR and IR

Where IR and PHR fundamentally intersect is in a seemingly shared desire to generate “real-world” evidence—with many social realities unfolding. Both can be viewed as a response to the limitations of the one-way pipeline model of transferring evidence-based knowledge from researcher to end-users such as community members. Without an understanding of community needs, the uptake of such knowledge can fall short. If the ultimate goal is therefore to understand and characterize these real-world and dynamic (as opposed to linear) contextual conditions under which different social actors operate and to improve knowledge use, then PHR is necessary for all implementation research efforts. Shirk et al. [22] argue that outcomes are affected by the degree and quality of public participation as negotiated at the outset. The research question or implementation problem is the starting point for implementation research and related methods. For example, the degree of intervention acceptability and appropriateness during implementation are key topics for interrogation in IR, requiring a robust account of the intervention embedded in its context and the use of participatory approaches [1, 23].

PHR and IR also intersect given the increasing imperative of better accounting for context to improve understanding of how and for whom an intervention works and to explain variations in implementation [24]. Researchers are being encouraged to better theorize about and measure context rather than treating it as something that is static and must be controlled for or as rationale for an intervention's implementation failure [25–27]. The ways in which PHR can best inform IR therefore concern who is involved and engaged in identifying salient dimensions of context and how these may drive inequities or create barriers to or enablers of change [3, 28]. The notion of adaptation of the intervention to context is also noteworthy. This is a feature of IR, although preoccupations with questions of fidelity also present in IR may limit the extent to which consideration is given to adaptive designs [2, 28]. To date, there has been a tendency for the salient dimensions of context and the nuanced characteristics of that context to be defined by researchers, sometimes in a way that is bereft of any authentic community engagement processes [29]. PHR approaches have also been promoted as a strategy to reduce health inequities [30], given some evidence that these approaches can improve social capital and cohesion [30]. The use of PHR approaches could help in better elucidating how an intervention adapts to its context throughout implementation and with what health and health equity impacts. These approaches can inform what the intervention actually entails, how it interacts with context in compatible and incompatible ways, how and why it changes over time, and “who” is affected disproportionately by social and health inequities potentially arising from the intervention's implementation [31]; they can also help better characterize and contextualize changes in coverage and benefits for intended participants [1]. Another area for further exploration might be how PHR can contribute knowledge about why the “fidelity” of an intervention is not retained or how and why interventions are adapted when implemented in a different setting.

### 3.4. Who Are the “Participants” in IR Studies and Is Community Engagement Always Necessary in IR or Not?

At its core, PHR requires the democratic participation of different actors (which vary depending on the intervention and the context)—researchers, policy-makers, health system practitioners, community members, citizens, and so on. “Who” participates, the extent to which they participate, and how and when they participate are all clearly pertinent to the operationalization of PHR approaches. This leads us to also ask whether or not community engagement (and also what is meant by community) is always necessary in IR. As described above, who is engaged and the nature and the extent of community engagement are points of divergence between PHR and IR. While considered an essential element of PHR, in IR studies, community engagement is not always considered essential and how community engagement is operationalized varies considerably. With PHR, we are most often referring to the main beneficiary of the intervention (e.g., community members, target population), while, in IR, the engagement may be policy stakeholders and those responsible for delivering the intervention. Second, the timing and frequency of that engagement also vary considerably between PHR and IR.

### 3.5. What Are the Possible Unintended Consequences of Participatory Processes in the Context of IR Studies?

Negotiating community and research perspectives and interests present ongoing conundrums in the application of participatory approaches in research, including but not limited to IR studies. Different career and cultural imperatives between academic and community partners require open debate and transparent discussion throughout the research process [32, p. 300]. “Involving people in decisions that affect them is justified both by ethical and political arguments and by instrumental arguments asserting that involvement will lead to decisions more relevant to the people being served” [33, p. 6]. While encouraging community engagement through the use of participatory processes comes with certain advantages, it can also present risks of potential harm and unintended consequences. Authentic engagement of communities does not always transpire in all research endeavours, let alone in implementation research. Engagement in research can make communities more vulnerable. Without the necessary safeguards, such as mechanisms of co-governance that ensure transparency and accountability in decision-making, communities can inadvertently become the objects of research agendas to the benefit of others. Katz et al. [34] documented harms related to, for example, delegated control (e.g., participatory process perpetuates status quo of neoliberalism, colonialism, and/or racism), demobilization (e.g., participant burn-out), or sanctions (e.g., participant resistance results in loss of paid work). These authors concluded that participation was often discontinued at implementation, making it challenging for communities to hold the conveners accountable, further underscoring differences in power relations [34]. If “communities” do know best and are well-positioned to contextualize an intervention by “integrating cultural values and practices to enhance sustainability when grant funding ends,” they also need to be valued as equal contributors in the production of knowledge [15]. This implies that, throughout the research, all partners be engaged in how the process is governed, recognizing the unique strengths that each can contribute while mitigating any potential harms from participation. Let us take the example of North-South partnerships in global health research.

#### 3.5.1. Power Relations between the “Participants” in the Research Process

The development and sustainability of Canada and low- and middle-income country (LMIC) partnerships that are egalitarian have been an objective of research funding in global health such as Canada's former Global Health Research Initiative [35]. It is, however, no easy feat and may in fact not be fully realized in practice given preexisting power asymmetries within teams, including those between researchers from different disciplines and decision-makers and communities, and across country contexts. Not only are preexisting power differences challenging to overcome in North-South partnerships, but they can also inadvertently undermine the ability to achieve an equitable orientation in research programming [36]. In an analysis of a Canadian-led global health research funding program, the authors argued that “the donor's finance and grant administration systems favored lower-risk Canadian institutions over generally higher-risk LMIC grantees and placed less restrictions on Canadian institutions than it did on LMIC ones” [36, p8]. Although inequitable fiscal relationships may adversely affect partnerships, other features of international partnerships can thrive. For example, an analysis across several case studies of North-South partnerships found that the global health partnered research could result in thriving and impactful partnerships. These partnerships were characterized as follows: (1) long-term and sustainable North-South partnerships; (2) interdisciplinary responses to complex issues; (3) participatory action research that grounds the research in its context; and (4) research with a policy or practice impact orientation [37]. Bearing the above intersections between IR and PHR in mind, we now explore the role and selected practices of research funders, and in particular CIHR.

### 3.6. Role and Practices of Research Funders

Research funders such as CIHR can incentivize different types of research such as implementation research through priority-driven funding calls. They can also encourage the ways research should be conducted through the use of specific eligibility and peer review criteria. Funders can build incentives to help make participation more feasible and equitable through a policy of release time allowance for knowledge users such as community members and decision-makers [38]. Tables 1 and 2 feature illustrative examples from priority-driven CIHR initiatives where deliberate efforts were taken to create funding initiatives and related criteria that support specific aspects of implementation research/science. They do not reflect the full range of IR funding calls at CIHR. The Pathways example emphasizes authentic engagements with Aboriginal communities, guided in part by OCAP® (Ownership, Control, Access, and Possession) principles [39]. In contrast, the HIV example is focused on implementation science across settings such as communities, prisons, and so on. The global health funding opportunities reflect an example to support IR on different health issues (e.g., maternal and child health, chronic diseases), across different disciplines, countries/regions (e.g., Sub-Saharan Africa), and sectors (e.g., health systems strengthening orientation). Through the use of criteria, funders such as CIHR are stipulating who should be involved in setting the overall priorities for the research and governing its implementation and thus implicitly encouraging the use of PHR methods without actually dictating which methods should be applied. Nevertheless, for some initiatives, a wide range of appropriate research designs and disciplines that may be considered are encouraged to pave the way for applying PHR approaches. In the context of research with vulnerable populations in Canada (e.g., Aboriginal communities) and in low- and middle-income countries where there is a history of misconduct in research perpetuating colonial practices, specific criteria may be even more essential. These need to be complemented by other mechanisms (e.g., guidelines, community ethics boards) to help ensure the ethical conduct of research.

## 4. Discussion

What remains an open question is the extent to which these funding incentives are having the intended impact—in terms of the degree and quality of participatory research processes and achieving meaningful results for communities and other participants in IR such as policy-makers and practitioners. The above featured examples raise important considerations for the monitoring and evaluation of participatory research processes in the context of research investments. Research funders such as CIHR use explicit eligibility and evaluation criteria to “steer” who ought to be involved in the research process, the approach to engagement, and how and the extent to which both the context and the intervention are described and are shaped by the engagement of different participants (e.g., communities, practitioners), while encouraging adequate documentation and reflexivity about ethical and equity considerations, including the ethical foundations for the interventions and the community engagement, extending what is typically considered in research ethics reviews [40]. These requirements can help set the stage for reporting requirements for researchers after funding and for assessing the impact of funders' practices.

There are of course limits to what funders can and cannot do to encourage researchers to use more participatory approaches in their research. Said funders could include grant writing proposal meetings where those most affected by the health inequities are brought into the discussions with researchers. They can then consider the option of engaging lay reviewers and asking them to specifically comment on the robustness of participant engagement approaches. For some grant reviews in the UK such as with the National Institute for Health Research (NIHR), funders are requiring a public engagement plan as part of the application for funding [41]. For such NIHR reviews, someone on the peer review committee (a lay person) is asked to review all grants to assess them and comment on their public engagement approaches.

## 5. Conclusion

In this paper, we describe the intersecting relationship between IR and PHR, identifying points of convergence and divergence and also highlighting how health research funders can advance both fields using illustrative examples from CIHR led/co-led funding initiatives. We conclude with a few areas requiring further exploration in implementation research and participatory health research and in the assessment of research funding agency practices.

Going forward, there is first a need to evaluate the science of funding to determine whether peer review criteria and other requirements influence researchers to expand relevant expertise on their team, to engage communities differently and use a wider range of appropriate and relevant participatory approaches. Relatedly, deliberate comparisons of participatory approaches across IR initiatives of various research funders are needed.

Second, researchers need to better describe the breadth and scope of participatory health approaches in reporting to funders and in the literature to enhance the evidence base of PHR in the context of IR. Jagosh et al. [42] conducted a systematic realist review to capture evidence about the benefits of participation and, in particular, which mechanisms and contextual features influence outcomes. They found that participatory research supports health by improving “research quality, empowerment, capacity building, sustainability, program extension, and unanticipated new activities” [42, p. 337]. Other scholars have shown through a meta-analysis that there is robust evidence that community engagement interventions positively impact health but perhaps not surprisingly no one model was found to be more effective than another [43]. Researchers need to be further encouraged to explicitly document the participatory health research approaches so the repertoire of methods that can be used is expanded and critiqued, any potentially harmful consequences are described (and better understood so they can be mitigated in the future), and the ways in which these processes do or do not enable implementation and scale-up of interventions are further elucidated.

## Figures and Tables

**Table 1:** *Eligibility and review criteria respecting who needs to be engaged in selected CIHR-funded initiatives on implementation research (IR).  Table 1* outlines excerpts from IR-focused initiatives led by/involving CIHR that favour the use of participatory health research (PHR). It focuses on the “*who*” needs to be engaged in IR. The eligibility criteria relate to who can lead the research, who must be involved in the research, and who can hold the funds. Eligibility is assessed first and if these criteria are not met, the application does not advance through peer review. The peer review criteria are used by reviewers to assess the proposals.

Funding initiative	Eligibility (who *must* lead and be involved in research)	Peer review criteria (how the research proposal is assessed)
CIHR Pathways to Health Equity for Aboriginal Peoples—Implementation Research Teams (component 2)	Independent researcher (Nominated Principal Applicant (PA)) At least one community member must be listed as a principal knowledge user Community-based policy/decision-maker expected to play key role in ensuring sound engagement strategies with communities.	Composition of team: “demonstrated experience working with Aboriginal communities in different contexts” Research and Community Engagement plan is culturally appropriate, gender sensitive, ethical, and methodologically sound Evidence of equitable and ethical partnerships with communities

Innovating for Maternal and Child Health—Implementation Research Teams (no longer publicly available) (CIHR, International Development Research Centre (lead) & Global Affairs Canada)	African researcher based in a targeted country (PA) Canadian researcher as a co-PA National level decision-maker as a co-PA (from same country as PA)	Team demonstrates a strong track record relevant to IR

HIV Implementation Science—Component 1	One knowledge user must be a community member with lived experience One knowledge user must be a decision-maker from service/policy-oriented organization.	Multidisciplinary, multisectoral team with expertise *relevant to involved communities/settings*; policy environments; disciplines relevant to IR

**Table 2:** *Evaluation criteria pertaining to the intervention and engagement approaches in CIHR-funding initiatives on implementation research*.  Table 2 features excerpts from implementation research-focused initiatives led by/involving CIHR. The evaluation criteria relate to the what (intervention) and related context(s) and how (research and community engagement approach), and they are used to assess the proposals. Criteria related to the research and community engagement approach signal how the funding opportunity was designed to ensure relevance and to try to mitigate harm or unintended consequences (i.e., culturally appropriate, collaboratively developed, and acceptable to communities; community-informed interventions; gender and equity considerations).

Funding initiative	Evaluation criteria	
	Description of the intervention and related context(s)	Research and community engagement approach
CIHR Pathways to Health Equity for Aboriginal Peoples Implementation Research Teams (component 2)	Addresses contextual factors (e.g., social, political, physical, and cultural), community engagement, and partnership processes thought to affect intervention implementation and scalability.	Governance plan for community engagement is well described, collaboratively developed with communities, culturally appropriate, and feasible Evidence that the proposed intervention is adaptable to different contexts, developed by and/or acceptable to communities, and potentially scalable Adaptation plans include *community-informed* intervention enhancements critical to improving effectiveness and scalability

Global Alliance for Chronic Diseases (lung disease call) (multiple funders)	Intervention relevant to the sociopolitical, cultural, legislative, and economic contexts of the study settings.	Inequities and equity gaps including gender taken into account in implementation strategy design (GACD, lung diseases)

Innovating for Maternal and Child Health (MCH)—Implementation Research Teams (no longer publicly available) (CIHR, International Development Research Centre (lead) & Global Affairs Canada)	Research relevant to countries' priorities.	Research driven by needs of communities, health care providers, program implementers, and policymakers
	Interventions to be studied demonstrate consideration for potential for scale-up to improve MCH.	Gender equality and equity considerations embedded throughout IR process Buy-in from decision-makers and other relevant stakeholders within/outside health sector

HIV Implementation Science—Component 1	Research questions, design, and methods are appropriate to assess interventions and heterogeneity of communities.	Feasible and appropriate plan for developing/extending relationships with communities Plan to collaborate with decision-makers and community partners to identify interventions and assess adaptation and contextual factors with relevance to implementation and scale-up
